# Composition within and between Languages in the Bilingual Mind: MEG Evidence from Korean/English Bilinguals

**DOI:** 10.1523/ENEURO.0084-21.2021

**Published:** 2021-11-01

**Authors:** Sarah F. Phillips, Liina Pylkkänen

**Affiliations:** 1 Department of Linguistics, New York University, New York, New York 10003-6636; 2 Department of Psychology, New York University, New York, New York 10003-6634; 3 NYUAD Research Institute, New York University Abu Dhabi, Saadiyat Island, Abu Dhabi, United Arab Emirates

**Keywords:** bilingualism, code switching, composition, language processing, LATL, MEG

## Abstract

The ability of the human brain to build complex expressions from simpler parts is fascinating, but the ability of the bilingual brain to do so is perhaps even more remarkable. When highly proficient bilinguals converse, they can fluidly switch from one language to another even inside sentences. Thus, they build expressions using words from more than one language. How are bilinguals able to compose words across different languages in real time? While robust evidence has implicated the left anterior temporal lobe (LATL) for the composition of words within one language, we do not know how the LATL, or other regions implicated for composition, operates when the language switches. We also do not know whether prefrontal regions associated with language control are recruited for language switching during composition. We addressed these questions with magnetoencephalography measurements in bilinguals who are fluent in two typologically distant languages, English and Korean. We observed early composition effects in the LATL at ∼200 ms that were unaffected by either language or orthography switching, which was also varied (Hangul vs Roman alphabet). Thus, the combinatory mechanism at 200 ms housed in the anterior temporal cortex appears blind to the language in which its input concepts are expressed. However, in later time windows, language and orthography switching interacted both in regions implicated for composition [LATL, ventromedial prefrontal cortex, left inferior frontal gyrus (LIFG)] as well as in regions associated with language control (ACC, LIFG). This establishes a starting point for understanding how bilingual brains code switch: words are initially combined without consideration of which language they come from, but language switching affects later processing.

## Significance Statement

Bilinguals are known to be able to switch languages in the middle of sentences. Despite an extensive literature investigating the role of prefrontal regions during language switching, little is known about how switching languages affects perisylvian regions implicated for language processing. Our research shows that the left anterior temporal lobe, a region widely implicated for composition, is insensitive to language switching as words are combined. This establishes a starting point for understanding how bilingual brains code switch, with ingredients from more than one language contributing to a complex structure and meaning.

## Introduction

How does the bilingual brain process code switching, that is, abrupt changes in language that require the brain to combine words or expressions from different languages? During production, bilinguals may switch languages when the desired expression is easier to access or better expressed in the other language. But for the comprehender, the switch can come as a surprise. In this work, we investigated language switching during composition from the comprehender’s perspective. How do bilingual brains rapidly accommodate language switches during comprehension, building complex structure and meaning from mixed-language ingredients with seeming ease? For any combinatory mechanism, understanding the constraints on its possible inputs is a fundamental part of characterizing its function. Here we tested whether neural activity sensitive to composition can operate with mixed-language input.

Current models of bilingual language processing largely focus on switching during production. Behavioral studies have found that bilinguals are slower in producing single words when they switch languages (Meuter and Allport, 1999; Costa and Santesteban, 2004). Because of these switch delays, models such as the Inhibitory Control Model of Green (1998) and the Mixed Language Frame Model of Myers-Scotton (1997) propose a language control mechanism for switching languages. Doing so explains observed production delays but provides little insight into online processing when bilinguals comprehend code-switching expressions.

When encountering a language switch inside a phrase or sentence, one possibility is that the combinatory mechanisms that operate within one language also operate across languages. Composition has been studied monolingually using both hemodynamic and electrophysiological measures, implicating the left anterior temporal lobe (LATL), left inferior parietal lobe (LIPL), ventromedial prefrontal cortex (vmPFC), and left inferior frontal gyrus (LIFG). Of these, the LATL has emerged as the most consistent correlate of composition across both imaging methods and modalities of language use (Pylkkänen, 2019). Early hemodynamic studies first showed increased LATL activity for composable sentences in comparison to noncomposable word lists (Mazoyer et al., 1993; Stowe et al., 1998), a finding later replicated with magnetoencephalography (MEG; Brennan and Pylkkänen, 2012; Hultén et al., 2019). This LATL composition effect is also observed for two-word phrases in written (Baron et al., 2010; Bemis and Pylkkänen, 2011; Westerlund et al., 2015) and auditory (Bemis and Pylkkänen, 2013; Sheng et al., 2019) comprehension cross-linguistically. Several other regions are also thought to contribute to combinatory processing: the LIPL, hypothesized to encode relational aspects of meaning (Boylan et al., 2017; Williams et al., 2017) and composition more generally (Binder and Desai, 2011; Bemis and Pylkkänen, 2013; Price et al., 2015, 2016; Graessner et al., 2021); the vmPFC, found sensitive to the semantic properties of combinatory expressions (Pylkkänen and McElree, 2007; Bemis and Pylkkänen, 2011; Pylkkänen et al., 2014; Blanco-Elorrieta et al., 2018); and the LIFG, associated with both long-distance dependencies (Stromswold et al., 1996; Leiken et al., 2015) and phrasal composition (Zaccarella and Friederici, 2015). The current study tests, for the first time, whether these regions (LATL, LIPL, vmPFC, LIFG) exhibit composition effects across code switches.

Another possibility is that the combinatory mechanisms that operate within one language either cannot operate across languages or cannot do so without the additional recruitment of prefrontal regions implicated for language control. The anterior cingulate cortex (ACC) has been implicated in various language-switching tasks, from passively listening to stories containing language switching (Abutalebi et al., 2007) to switching languages while naming objects (Garbin et al., 2011). In addition to the ACC, the dorsolateral prefrontal cortex (dlPFC) and LIFG have been reported during picture-naming tasks that involve language switching (Hernandez et al., 2000, 2001) and reading code-switching sentences (Rossi et al., 2021). All three of these regions are included in language control network of Abutalebi and Green (2016), with each region likely responsible for different demands imposed by code switching (Seo et al., 2018). Because these language control regions have largely been identified using fMRI, the current study tested not only whether control regions are engaged during code-switched composition but also when code-switching elicits activity in these regions millisecond by millisecond, assessing the temporal ordering of potential composition and language control effects.

To measure the effects of basic composition and to test their modulation by language switching, we created a Korean/English bilingual version of the original Bemis and Pylkkänen (2011) basic composition paradigm. The original paradigm measured the neural activity of nouns that were preceded by either a combining adjective in a composition task (e.g., “red boat”) or a noncombining noun in a list task (e.g., “cup boat”). They observed that combinatory phrases elicited greater activity than noncombinatory lists (i.e., composition effects) in the LATL and vmPFC. However, adjective–noun combinations would have been problematic for our study because of the properties of Korean morphology (see Materials and Methods). From the various alternatives we considered, the best structural parallelism between Korean and English was achieved by subject–verb combinations, which have also engaged the LATL in prior work (Zhang and Pylkkänen, 2018b; Kim and Pylkkänen, 2021). Our stimuli thus consisted of two-word combinatory sentences (e.g., “icicles melt”) that varied whether the language switched or not between the subject and verb. Noncombinatory controls were created by substituting subjects with verbs, yielding two-verb lists whose list interpretations we reinforced by presenting them in a LIST task (where participants were asked whether a subsequent picture matched one of the two verbs presented, making composed interpretations highly unlikely) as in the study by Bemis and Pylkkänen (2011). Finally, our design contained a manipulation of orthography switching, given that Korean can be written in Hangul and the Roman alphabet. With this approach, we addressed how combinatory processing and language control regions behave during the comprehension of code switches. Our findings suggest that the LATL houses a combinatory mechanism that composes single-language and mixed-language inputs with comparable ease.

## Materials and Methods

### Participants

Korean/English bilinguals were recruited through flyers and word of mouth in the New York City area. Twenty Korean/English bilingual adults (16 female, 4 male; mean age = 25.60 years, SD = 8.44 years) participated in the study. All participants reported being right handed and neurologically intact with normal or corrected-to-normal vision. They also completed the LEAP-Q (Language Experience and Proficiency Questionnaire; Marian et al., 2007), which provided insights into their language history and proficiencies. The age at which participants began acquiring Korean was quite consistent (mean age = 0.60 years, SD = 0.99), while the age at which they began acquiring English varied more (mean age = 6.45 years, SD = 4.89). Our participants reported reading more in English (mean time = 69.2%, SD = 23.0%) than in Korean (mean time = 31.2%, SD = 22.4%) on a typical day, though they reported comparably high proficiency ratings in reading English (mean rating = 9.50 of 10, SD = 1.05) and Korean (mean rating = 8.70 of 10, SD = 1.84). They also reported being exposed to English more (mean = 66.5%, SD = 18.9%) than Korean (mean = 31.4%, SD = 17.7%) on a typical day. Because the participants reported high proficiencies in both languages despite spending more of their typical day engaging in English than Korean, we considered the participants to be highly proficient in both languages. This is further supported by the results of their lexical confidence questionnaires, as they reported high familiarity with the English (mean = 9.97, SD = 0.27) and Korean (mean = 9.70, SD = 1.34) lexical items used in the study.

### Stimuli and experimental design

**Figure 1.**
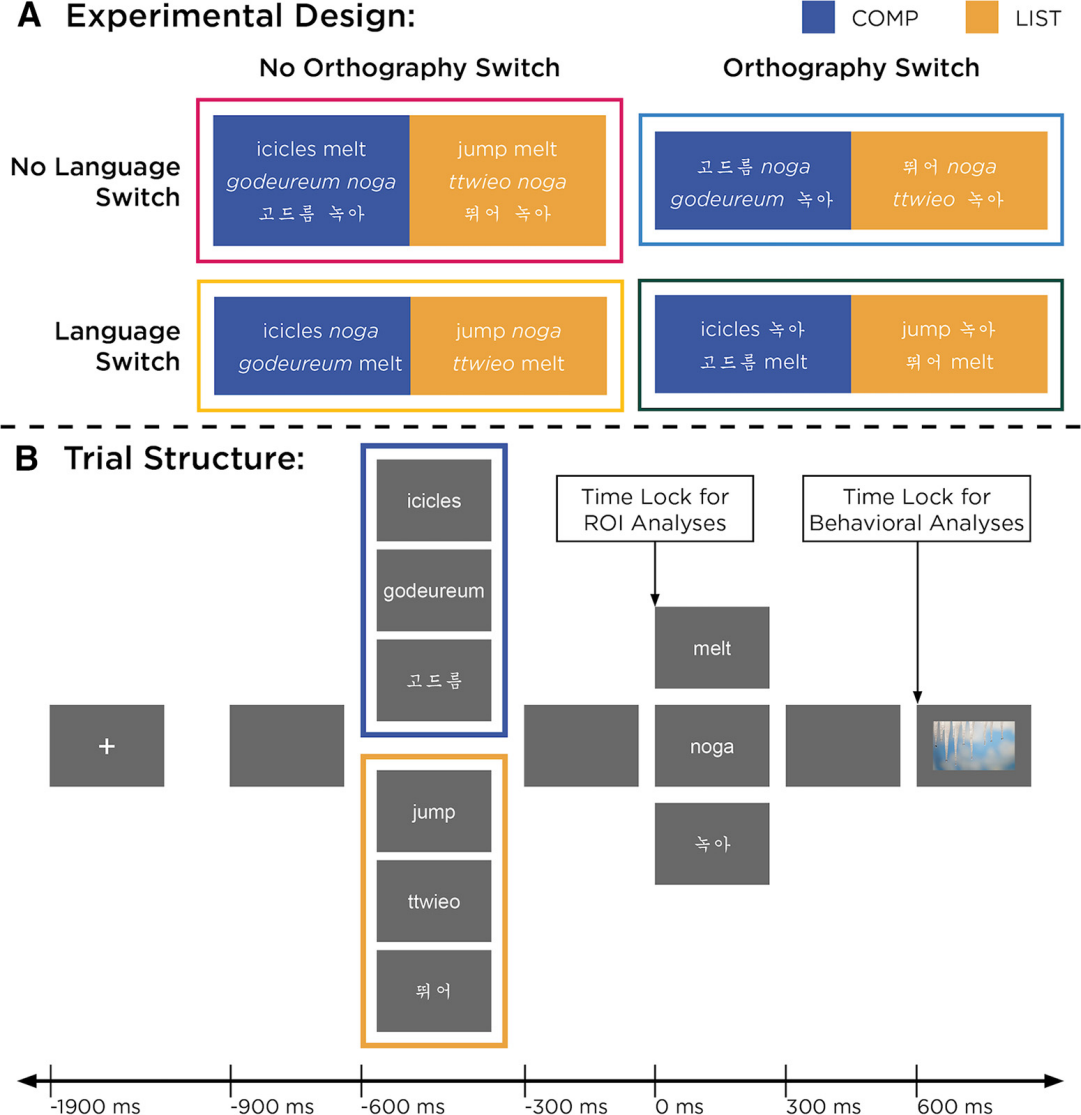
***A***, Stimuli varied by three factors. The first factor was composability, where two-word items that composed into short sentences (i.e., COMP items) are marked in blue and two-word items that did not compose (i.e., LIST items) are marked in gold. The second factor was language switching (Switch, No-Switch); and the third factor was orthography switching (Switch, No-Switch). All example stimuli express “icicles melt” but illustrate the varied presentations by our conditions. ***B***, Trials were blocked by stimulus type (COMP, LIST). Although stimulus presentation was consistent across all trials, the behavioral task differed between the two block types. For each COMP trial, participants indicated whether the picture matched or did not match the preceding two-word expression; for each LIST trial, participants indicated whether the picture matched one of the two preceding words or did not match either word. Pictures were balanced across trials for whether they matched/mismatched.

To investigate the effect of language switching on basic composition, we first adapted the two-word composition paradigm of Bemis and Pylkkänen (2011), an attempt to control for the linguistic differences between English and Korean, and then crossed composability (COMP, LIST) with language switching (switch vs no-switch) and orthography switching (switch vs no-switch; Fig. 1). The original Bemis and Pylkkänen (2011) paradigm used adjective–noun phrases (e.g., red boat) for their two-word composition (COMP) trials, but Korean adjectives are typically expressed as verbs and must be inflected with an additional morpheme (-n/neun) to come before a noun. We thus decided to use two-word sentences, consisting of a subject and an intransitive verb (e.g., icicles melt), in the combinatory trials (COMP) instead of adjective–noun phrases. We then created verb–verb lists from the verbs used for the COMP trials, functioning as our two-word noncombinatory LIST controls (e.g., jump melt). Doing so maintained syntactic and semantic congruence among COMP and LIST items such that language and/or orthography switching effects observed would not be confounded with morphologic effects, such as morphologic decomposition (Solomyak and Marantz, 2010).

Selecting lexical items for our stimuli involved detailed consideration because of the lexical differences between English and Korean. For the verbs, we selected commonly occurring verbs that behaved as intransitives in both English and Korean to avoid word order differences. In typical declarative sentences, English presents an object after the verb, whereas Korean presents an object before the verb. Using intransitive verbs, which do not permit objects, allowed us to maintain congruent grammatical structures between English and Korean. For the subjects, we needed single-word, concrete nouns that commonly occurred in both languages. We chose bare plural nouns in English to avoid needing a determiner (e.g., a/an) and then identified their Korean equivalents. Doing so was easier since Korean nouns are ambiguous regarding number, meaning they can be interpreted as singular or plural when bare (e.g., “godeureum” can be interpreted as “icicle” or “icicles”). However, the English nouns varied by whether they are mass nouns (i.e., nouns that cannot be counted and therefore do not straightforwardly pluralize) or count nouns (i.e., nouns that can be counted and therefore do have a plural form). Given that morphologic decomposition (Solomyak and Marantz, 2010) and the mass/count distinction (Domahs et al., 2012; Williams, 2018) both elicit effects in temporal regions during online processing, nouns were balanced for morphologic irregularity/regularity (i.e., whether plurality is marked using “-s” or some irregular form including no marking) in English. By composing nonsingular nouns with semantically plausible intransitive verbs, all COMP stimulus items thus read as generic statements, expressions that describe characteristics of a kind rather than a select group of individuals [e.g., “icicles melt” means that all icicles (have the ability to) melt; Krifka and Gerstner, 1987; Chierchia, 1998].

The fact that English is typically read in the Roman alphabet and Korean in Hangul led us to include orthography switching as a factor in our design. To deconfound language switching from orthography switching, Korean lexical items were presented in both Hangul and the Roman alphabet. The additional manipulation was fairly natural since South Korea has a history of Romanizing, that is, transcribing the Korean language into the Roman alphabet (http://monthly.chosun.com/client/news/print.asp?ctcd=&nNewsNumb=200009100029). Because of this history and that many first-generation Korean Americans immigrate from South Korea (and not North Korea or China), reading Korean in the Roman alphabet was expected to be fairly natural for our subjects. In contrast, English loan words are often kept in the Roman alphabet and not transcribed into Hangul (despite also being a phonetically based writing system), and therefore we did not transcribe our English stimuli into Hangul.

To ensure that COMP and LIST items were compositional and noncompositional, respectively, an acceptability judgment task was designed. English speakers, Korean speakers, and Korean-English bilinguals not participating in the MEG study were presented with two words and then asked how well the two words combined into a single expression using a 7-point scale (−3, not well at all; 0, neutral; 3, very well). English speakers were presented with English-only COMP and LIST items; Korean speakers who did not identify as Korean/English bilingual were presented with Korean-only COMP and LIST items; and Korean/English bilinguals were presented with the language-switching COMP and LIST items. COMP and LIST items were randomly presented to each participant group. Responses from 165 English speakers were collected for the English-only items using Amazon Mechanical Turk (MTurk). Results emerged as predicted where COMP items scored high (mean = 2.22, SD = 0.53) and LIST items low (mean = −1.90, SD = 0.53). However, native Korean speakers and Korean-English bilinguals were difficult to recruit through MTurk. Thus, 20 native Korean speakers and 10 Korean-English bilinguals were recruited through word of mouth and instead gave informal acceptability judgments (Sprouse et al., 2013). Those results patterned like the MTurk results for their English equivalents, where well formedness was considered “well” for COMP items and “not well” for LIST items even across language-switching items.

As in the original study by Bemis and Pylkkänen (2011), COMP and LIST stimulus items were blocked separately because they also differed in task: the COMP task was to match the mini-sentence to a subsequent picture, eliciting natural combinatory processing, while the LIST task required subjects to indicate whether either verb matched the subsequent picture. The reason for having different tasks for each stimulus type was to discourage participants from attempting to compose noncombinatory LIST lexical items together. Despite having tested the differences in composability between COMP and LIST items, people are known (at least anecdotally) to coerce interpretations out of implausible word combinations. Each block consisted of 40 trials, and there were nine blocks for each stimulus type (COMP, LIST). Block order was counterbalanced across participants. Trials in each block were randomized and balanced by our other two experimental factors, language switching and orthography switching. In other words, each block randomly presented trials that had no switching (e.g., English-English), language-only switching (e.g., English-Korean), orthography-only switching (e.g., Korean-한글), or both (language and orthography switching; e.g., English-한글). In each trial, participants were presented with a fixation cross, followed by each of the two words successively in a stimulus item. After the second word, participants were presented with an image. Participants indicated on a keypad with either their left forefinger for when the picture matched or left middle finger for when the picture did not match. Trials would only advance once participants pressed either button. Participants typically spent 50–60 min to complete all 720 trials.

### Procedure

Upon their arrival at the laboratory and after giving informed consent, participants filled out a handedness questionnaire (Oldfield, 1971) electronically as well as a modified version of the LEAP-Q questionnaire (Marian et al., 2007) on paper. Before the MEG recording, participants’ head shapes were 3D digitized to enable coregistration of the MEG data with the FreeSurfer average brain during analysis (CorTechs Labs and Massachusetts General Hospital (MGH)/Harvard Medical School(HMS)/ Massachusetts Institute of Technology (MIT) Athinoula A. Martinos Center for Biomedical Imaging, Charlestown, MA). The following two different digitizers were used: the first 13 participants’ head shapes were digitized using a Polhemus FASTRAK three-dimensional digitizer (because our original Polhemus FastSCAN II digital scanner was found nonfunctional when trying to scan our first participant); and the remaining 7 participants’ head shapes were digitized using our replacement Polhemus FastSCAN II digital scanner. All digitizations included information from approximately five marker coil placements on each participant’s head and the positions of three fiducial landmarks (nasion, left tragus, and right tragus).

After head-shape digitization, participants engaged in a practice version of the experiment presented using Psychopy (version 3.0.0b11) on a 2018 MacBook Pro. Trial structures were consistent for the practice and the actual experiment. Each trial started with a fixation cross that lasted 1 s, followed by a blank screen for 300 ms. Then, the first word of a stimulus item, a blank screen, and the second word of the same stimulus item were each presented for 300 ms. After another blank screen for 300 ms following the second word, participants were presented with an image for the behavioral task. These images were balanced and randomized within and across blocks for whether they matched/mismatched with the verbal stimuli. Participants were unable to advance through trials until they responded on the keypad using either the left fore or middle finger. All practice trials used stimulus items not used in the experiment.

Participants who completed the practice and felt comfortable with the task were then guided into the magnetically shielded room hosting the MEG machine. Marker coils were placed onto participants at the points marked during the head-digitization process, and then participants were laid into the MEG helmet. MEG recordings were collected as participants completed the experiment. After the recordings were completed, participants were asked to fill out a lexical confidence questionnaire that inquired how familiar they were with the words used during the study on a scale from 0 (not familiar at all) to 10 (highly familiar). Eighteen of the 20 participants completed this final questionnaire. English lexical items were displayed in the Roman alphabet, and Korean lexical items were displayed in Hangul. Overall, participants spent ∼1 h and 40 min in the laboratory.

### MEG data acquisition and preprocessing

All MEG data were recorded using a whole-head, 157-channel axial gradiometer system (Kanazawa Institute of Technology, Nonoichi, Japan) at a sampling rate of 1000 Hz with a low-pass filter at 200 Hz and a notch filter at 60 Hz. Recordings were noise reduced by the continuously adjusted least-squares (CALM) method (Adachi et al., 2001) and filtered with a 1 Hz high-pass filter using the MEG160 software included with the MEG system. After noise reduction, recordings were preprocessed using MNE-Python (Gramfort et al., 2013) and Eelbrain (version 0.29.8; https://doi.org/10.5281/zenodo.2552354). Channels that were flat lined were marked and removed first (averaging six channels per participant). Then, epochs were created from 700 ms before onset of the second word (or 100 ms before onset of the first word) to 600 ms after onset of the second word (or at the onset of the presentation of the image). Epochs that exceeded 3000 fT and components containing artifacts (e.g., eye blinking, heart beats) identified using independent component analysis were rejected. Doing so excluded ∼3.76% of the total number of trials completed by each participant. Because the design itself allows an imbalance in the number of epochs per condition (60 COMP and 60 LIST “No Switch” trials; 40 COMP and 40 LIST “Orthography Switch” trials; 40 COMP and 40 LIST “Language Switch” trials; and 40 COMP and 40 LIST “Language & Orthography Switch” trials), which becomes more imbalanced during epoch rejection, epochs are subsequently balanced by keeping the first × number of epochs for each condition. This was done to be able to run a repeated-measures ANOVA for the analyses. All remaining epochs, which were ∼93.42% of epochs for each participant per condition, were baseline corrected using 700–600 ms before onset of the second word (or the first 100 ms before onset of the first word).

The distribution of neural activity across the source space was estimated using MNEs (Hämäläinen and Ilmoniemi, 1994). Cortical surfaces were constructed by morphing and projecting mapping of an average brain from FreeSurfer (Dale and Sereno, 1993) to the head-shape digitizations collected from each participant. A total of 5124 points (or dipoles) were generated onto the source space for each participant, and the forward solution, the magnetic fields calculated from the sensor recordings with respect to the 5124 dipoles, was calculated using the boundary-element model (BEM). To eliminate any additional noise recorded by the sensors, the noise covariance matrix was then calculated using the first 100 ms of each epoch (which was also 100 ms before onset of the first word presented). Using the calculated forward solution and noise covariance matrix, an inverse solution was computed using a fixed orientation that restricts the orientation of the dipoles to be orthogonal to the surface of the projected cortex (Lin et al., 2006). Finally, the minimum norm current estimates, which were produced from calculating the root-means-square of the activity from a subset of the fixed dipoles, were converted to dynamic statistical parametric maps (dSPMs) for visualizing activity in both space and time.

### Behavioral data analyses

Early behavioral studies investigating language switching have found delayed responses when participants must switch languages between trials (Meuter and Allport, 1999; Costa and Santesteban, 2004). These studies formed the foundation for hypotheses proposing a role for prefrontal regions in language control. Unfortunately, response times (i.e., how long participants took to press a button once presented with an image in each trial) and accuracy (whether participants correctly performed the task in each trial) were recorded from only 13 of the 20 participants because of technical issues. Response times of inaccurate responses and times exceeded 2.5 SDs from the overall mean were excluded. The resultant response times and accuracy counts were then subjected to 2 × 2 × 2 ANOVAs comparing composition, language switching, and orthography switching. Doing so determined whether our manipulations affected participants’ performance on the picture-matching tasks. Both behavioral measures were analyzed in R (version 4.0.3) and RStudio (version 1.3.1093).

### Region of interest analyses

To identify the effects of composition, language switching, and orthography switching, we searched for clusters of time points during which our manipulations significantly affected activation patterns in each region of interest (ROI). For ROI definition, we used Brodmann areas [BAs; PALS_B12_Brodmann annotation file with FreeSurfer (http://surfer.nmr.mgh.harvard.edu/); Dale et al., 1999] based on results from relevant, previous studies. For regions implicated for composition, we used BA38 for the LATL (Bemis and Pylkkänen, 2011; Westerlund et al., 2015; Zhang and Pylkkänen, 2015), BA11 for vmPFC (Bemis and Pylkkänen, 2011), BA44 for the LIFG (Hernandez et al., 2001; Friederici and Kotz, 2003; Zaccarella and Friederici, 2015) and BAs 39 and 40 for the LIPL (Williams et al., 2017). Given the limited spatial resolution of MEG, we only chose one posterior ROI, the LIPL, and decided to only extend the analysis to adjacent posterior temporal cortex should the LIPL show effects. For language control ROIs, we followed Blanco-Elorrieta and Pylkkänen (2016) and chose BAs 24, 32, and 33 for the ACC and BAs 9, 10, and 46 for the dlPFC. To assess the lateralization of any observed effects, the right hemisphere homologs of all the ROIs were also analyzed.

The sources within each ROI were averaged and significant clusters of time points in ROI activity, corrected for multiple comparisons, were identified using a cluster-based permutation test (Maris and Oostenveld, 2007). At each time point, a 2 × 2 × 2 repeated-measures ANOVA (composition × language switching × orthography switching) was performed to calculate an *F* test statistic. Temporal clusters were identified when a set of contiguous time points, extending longer than 25 ms, had *F* statistics that exceeded the critical α-value of 0.05, and the *F* values of the time points in the cluster were then summed to yield a test statistic for the cluster. A total of 10,000 permutations were then generated for each ROI, randomly shuffling condition labels for each participant. We created a distribution of these 10,000 cluster permutations and compared the permuted clusters to the statistics of the actual clusters observed. The *p* values generated for each cluster within an ROI reflect the proportion of permuted clusters with sizes larger than the actual cluster observed within each designated time window. Activity elicited by the first word and the second word were analyzed separately: the first word time window was from 600 to 0 ms before onset of the second word (or −600 to 0 ms), and the second word time window was from 0 to 600 ms. The output *p* values of the within-regions permutation procedure were corrected across the other functionally relevant regions (combinatory or control) using the false discovery rate procedure (Benjamini and Hochberg, 1995) in R (version 4.0.3) and RStudio (version 1.3.1093) with a critical value of 0.05.

## Results

### Behavioral results

**Figure 2.**
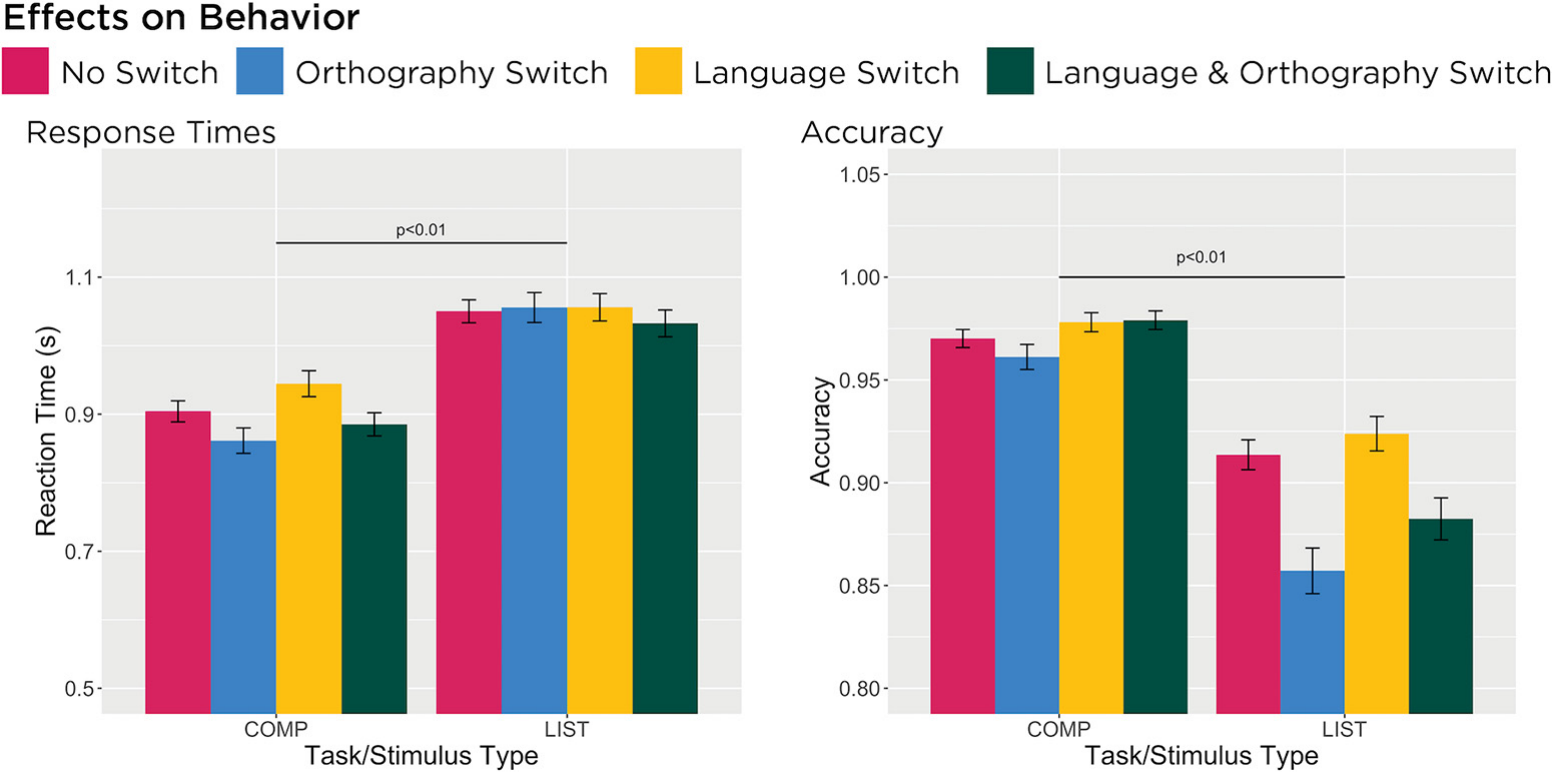
Bar graphs represent average response times and average proportion of correct answers, respectively, by each condition, and error bars represent the SEM. Participants were significantly faster and more accurate completing the COMP task than the LIST task (*p* < 0.01). They were also significantly more accurate (*p* < 0.01) when the orthography did not switch in the LIST task.

Averaged response times and averaged proportion of correct responses are depicted in Figure 2. For the response times, our analyses found that participants were faster identifying whether pictures matched a two-word sentence (COMP) than one word of a two-word list (LIST; *F*_(1,8)_ = 27.28, *p* < 0.01, η_p_^2^ = 0.77), which is consistent with the original study by Bemis and Pylkkänen (2011) and subsequent work. Fixing effects for task, neither language switching (*F*_(1,8)_ = 0.18, *p* = 0.68, η_p_^2^ = 0.02) nor orthography switching (*F*_(1,8)_ = 0.003, *p* = 0.96, η_p_^2^ < 0.01) affected participants’ response times. However, performing a Tukey’s HSD *post hoc* test revealed that participants were significantly faster when the orthography switched specifically in the COMP task (*p* = 0.01). Participants were overall highly accurate (mean = 0.93, SD = 0.25), but our analyses revealed that participants were more accurate during the COMP task than the LIST task (*F*_(1,11)_ = 181.12, *p* < 0.01, η_p_^2^ = 0.94). Fixing effects for task, they were also more accurate when the orthography did not switch (*F*_(1,11)_ = 21.13, *p* < 0.01, η_p_^2^ = 0.66). Performing a Tukey’s HSD *post hoc* test revealed that orthography switching significantly affected participants performance in the LIST task (*p* < 0.01) but not the COMP task (*p* = 0.93). Overall, our results reveal no behavioral effects of language switching but instead an effect of orthography switching on participants’ response times in the COMP task and accuracy in the LIST task.

### Combinatory effects

**Figure 3.**
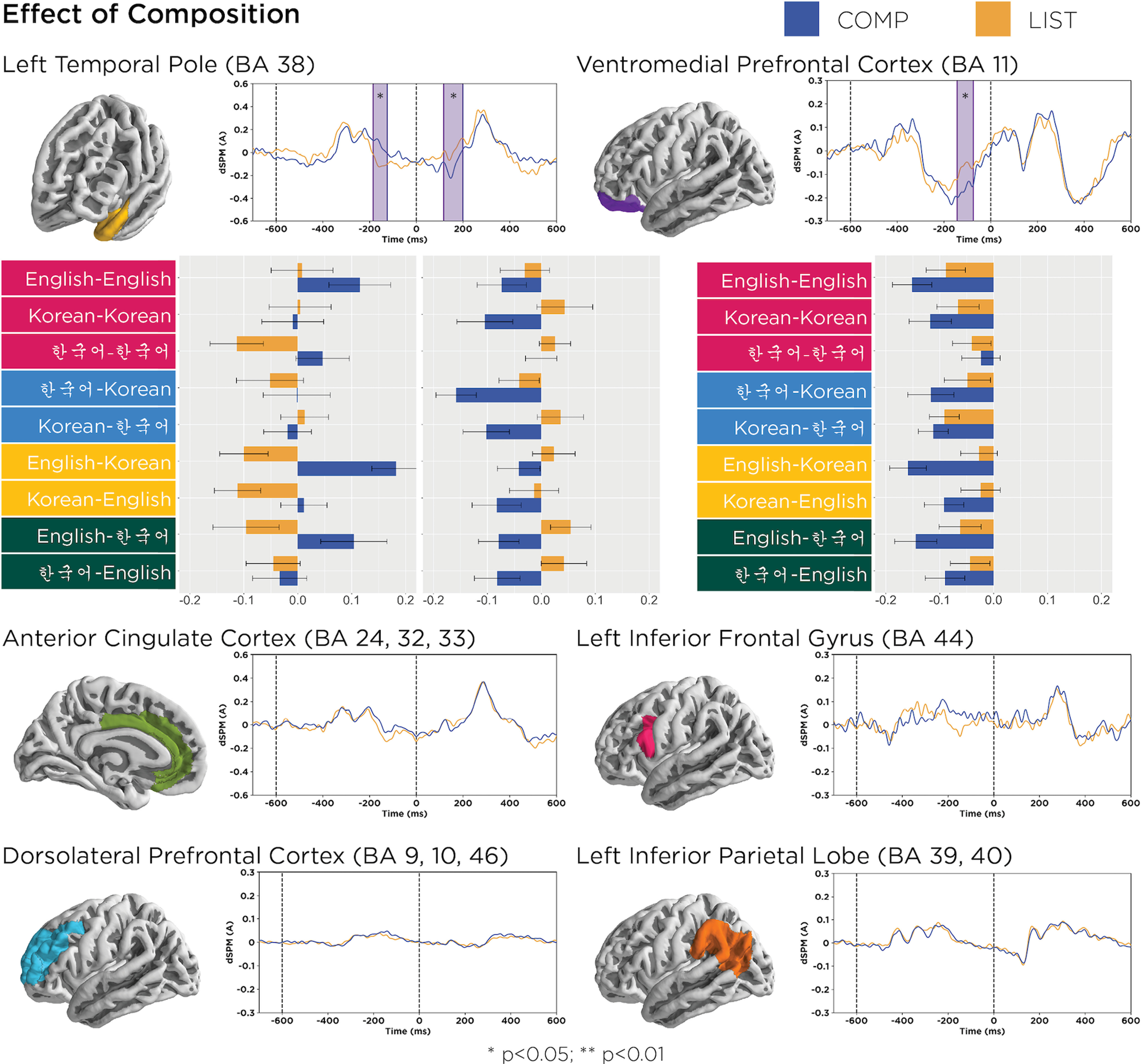
Bar graphs provide mean activation values in each significant time window for each language pair, and error bars represent pooled SEs. A composition effect was observed in the left temporal pole between 117 and 199 ms (*p* = 0.04), which did not interact with language or orthography switching. Examining each language pair revealed a similar pattern in the second time window compared with the effect observed in the full design. Effects were also observed in the first time window in the LATL, where nouns elicited more positive activity than verbs, and in the left vmPFC, where nouns elicited more negative activity than verbs.

Of the “combinatory regions” (LATL, vmPFC, LIPL, LIFG), we expected composition to affect at least the LATL at ∼200 ms after the onset of the second word, as observed in prior work, but the main question was whether this effect would interact with language switching or orthography switching. Averaged waveforms across participants for each “combinatory ROI” are depicted in Figure 3.

Our analyses in the first time window revealed a main effect of composition in both the LATL (BA 38) and left vmPFC (BA 11). The nouns of the COMP trials elicited more positive activity, while verbs of the LIST trials elicited more negative activity, in the LATL at −185 to −125 ms (or 415–475 ms from onset; *p* = 0.05). In the vmPFC, first position COMP nouns elicited more negative activity than LIST verbs at −144 to 75 ms (or 456–525 ms from onset; *p* = 0.05). These findings before the onset of the second word could reflect either lexical category (noun vs verb) or, as also observed in the study by Neufeld et al. (2016), anticipatory effects of composition, since composition in our design was fully predictable (the conditions were both blocked and differed in lexical category in the first position). Analyses of the right hemisphere homologs in the first time window, after correcting for multiple comparisons, did not reveal any significant effects of composition.

Our analyses in the second time window revealed a main effect of composition only in the LATL at 117–199 ms (*p* = 0.04), unqualified by interactions. Despite the COMP verbs being more predictable than LIST verbs in this time window, the COMP verbs elicited greater negative activity than LIST verbs. This suggests that the left temporal pole engages in early combinatory processing without sensitivity to language switching or orthography switching. Though there were no interactions, Figure 3 shows the pairwise comparisons of COMP versus LIST for each of the individual language pairs (e.g., “Roman-Hangul” represents trials where the first word was a Korean lexical item presented in the Roman alphabet and the second word was a Korean lexical item presented in Hangul), plotting the means over the interval of the significant main effect, and indeed all pairs pattern similarly. Analysis of the right hemisphere homologs in the second time window, after correcting for multiple comparisons, also did not reveal any significant effects of composition.

### Switching effects: interaction between language and orthography switching

**Figure 4.**
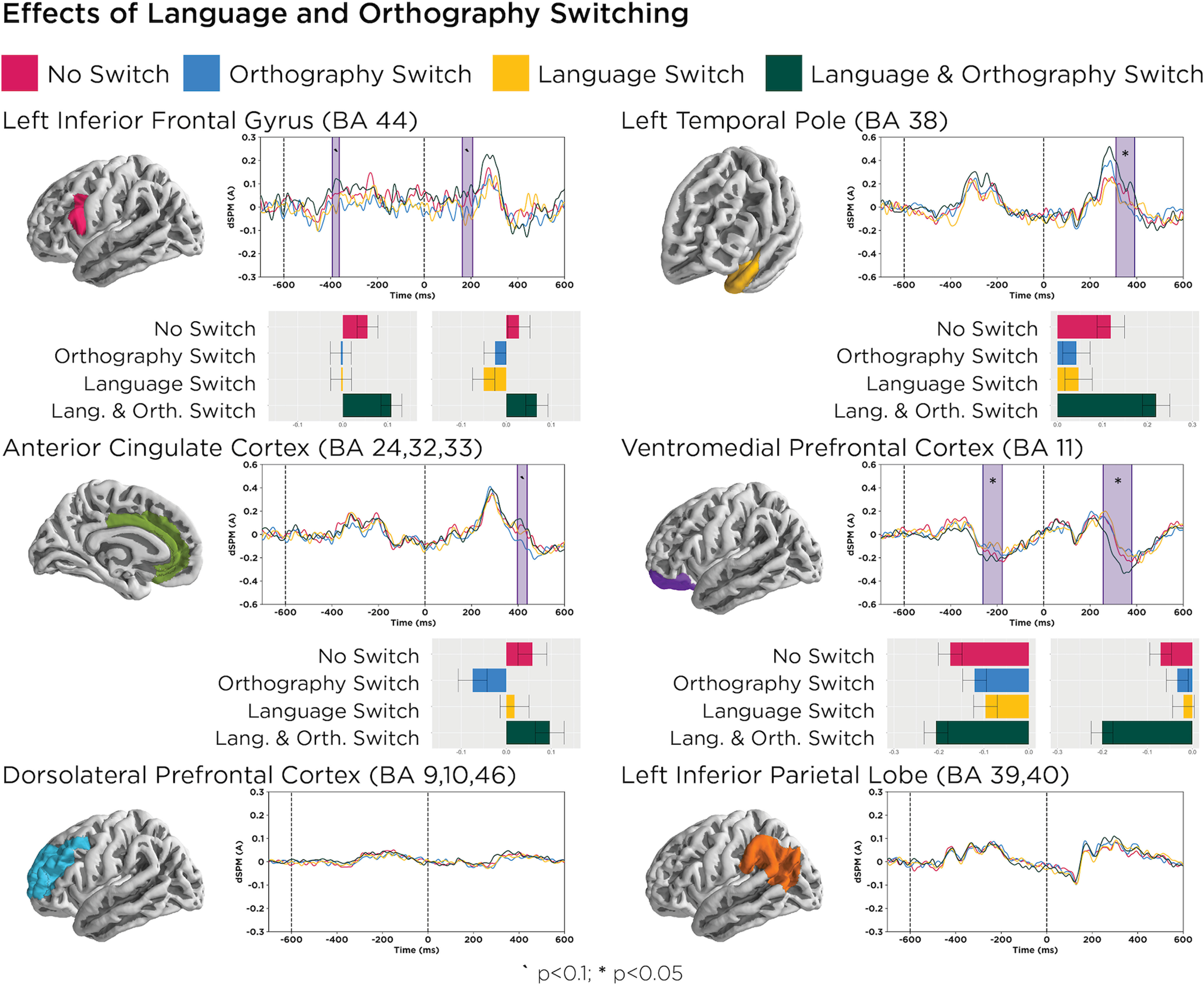
Bar graphs provide mean activation values in each significant time window, and error bars represent pooled SEs. While a main effect of language switching was not observed, correspondence between language and orthography switching (i.e., either both language and orthography switched or neither language nor orthography switched) elicited trending effects in the left ACC (*p* = 0.06) and LIFG (*p* = 0.06) as well as significant effects in the LATL (*p* = 0.02) and left vmPFC (*p* = 0.01).

For the language control regions (ACC, dlPFC, and LIFG), our main question was whether they would show switching effects when the switch occurs in the middle of a combinatory expression. Averaged waveforms across participants for both language control and combinatory processing ROIs are depicted in Figure 4. Responses to the second word did not show effects of language switching in any of our language control regions.

However, an interaction pattern between language switching and orthography switching was observed not only in the “control regions” (ACC and LIFG), but also in the combinatory regions (LATL and vmPFC) in the left hemisphere. Specifically, in all of these regions, the following two conditions patterned together: the condition in which nothing was switching (neither language nor orthography); and the condition in which both language and orthography switched, with the latter always eliciting the largest amplitudes. This pattern could indicate sensitivity to a correspondence between language switching and orthography switching: switching orthography signals, switching languages, and maintaining an orthography signal maintaining the same language. The left ACC and LIFG results were marginal (both *p* values* *=* *0.06) and were driven by more positive activity when both the language and orthography either switched or did not switch (ACC, 397–439 ms; LIFG, 163–207 ms). The LATL showed more positive-going activity at 311–391 ms (*p* = 0.02) when both language and orthography switched, while the left vmPFC showed more negative-going activity when both language and orthography switched at 256–379 ms (*p* = 0.01). Analysis of the right homologs revealed a similar, but inverted, pattern in the right vmPFC between 478 and 534 ms (*p* = 0.06), where language-only and orthography-only switches elicited more negative activity.

## Discussion

### No effect of language switching on composition in the LATL

This work investigated how bilingual brains combine words presented in two different languages, a striking skill that comes naturally for most bilinguals. We tested both for effects of composition and for effects of language switching using a well established two-word composition paradigm. We found that the LATL engaged in combinatory processing unencumbered by language switching. Combinatory effects in the LATL have previously been shown to reflect conceptual as opposed to syntactic or semantic aspects of composition (Westerlund et al., 2015; Zhang and Pylkkänen, 2015, 2018a). Thus, the LATL appears to be a conceptual hub sensitive to composition that does not require its input concepts to come from the same language.

It was already known that the sensitivity of LATL to composition is modality general (Pylkkänen, 2019), but the finding that it is also language general, insensitive to switches, importantly adds to our functional understanding of this region. The earliness of the main effect of composition at 150–200 ms in particular suggests that the combinatory mechanism in the LATL and the requisite semantic access operate seamlessly even when language is switching. In addition, the current results revealed LATL and vmPFC sensitivity to composition already before the onset of the second word, consistent with structure prediction (Neufeld et al., 2016), though interpretations in terms of lexical category (noun vs verb) cannot be ruled out. Prior fMRI studies have also reported the involvement of LATL during bilingual language comprehension (Perani et al., 1998; Abutalebi et al., 2007), but with a focus on language switching as the main manipulation. It has been argued that while similar regions are activated for the two languages of a bilingual, a possible neural signature of bilingualism is the increased recruitment of these shared regions compared with their monolingual counterparts (Kovelman et al., 2008). However, we did not collect data from English or Korean monolinguals to test whether this is supported in the LATL.

So far, the profile of the LATL lends support to a bilingual language processing model in which a single system of composition can apply cross-linguistically, similar to what MacSwan (2000) has argued. Additional support for this view comes from recent behavioral evidence by Declerck et al. (2020), who explored the so-called sentence-superiority effect (Snell and Grainger, 2017) in a bilingual code-switching setting. The authors presented bilinguals with four-word strings for 200 ms that were either grammatical code-switching sentences or scrambled language-switching word lists, and then asked the participants to correctly identify a word within the string. Responses were more accurate for target words in sentences than in word lists even when the sentences contained language switching, an effect that the authors dubbed as the “bilingual sentence superiority effect.” It is possible that this type of rapid sentence composition across languages is enabled by the LATL processes measured here.

However, in self-paced reading tasks, language switching has been reported as costly. For example, Dutch/English bilinguals were slower to advance words in self-paced reading when the language switched (Bultena et al., 2015). Similarly, Chinese/English bilinguals were slower at selecting a subsequent word in a MAZE task when the language (and orthography) switched (Wang, 2015). We cannot, however, with any confidence, attribute these effects to combinatory processing specifically. Our behavioral results found that Korean/English bilinguals were faster matching pictures to composable, orthography-switching sentences; but the LATL exhibited an effect of language–orthography correspondence in a later time window (311–391 ms) than the composition effect (117–199 ms). The results together suggest that other mechanisms activated later than early combinatory processing may be affected by language and/or orthography switching. For example, nothing rules out a lexical source for the observed effects, as proposed by MacSwan (2000).

Regarding possible alternative interpretations of our LATL findings, under the traditional assumption that less processing effort correlates with reduced neural activity, hypotheses in terms of lexical predictability, semantic relatedness, and task difficulty would all predict activity decreases for the combinatory stimuli compared with the list stimuli. Such a prediction is opposite from our observed effect (as also was the case in the study by Bemis and Pylkkänen, 2011). This is because our target verbs were more predictable and more semantically related to their context words in the COMP task than in the LIST task, and the LIST task was also the harder task given that it included an additional working memory component (keeping the first word in mind is less natural in the LIST task than in the COMP task). Thus, our design was biased against observing the desired effect. One interesting question for the future is whether and how the combinatory LATL activity relates to the phrase- and sentence-level effects obtained in the frequency domain in the study by Ding et al. (2016); it is possible that the LATL could be one contributor to such effects.

### Interactions between language switching and orthography switching

Language switching elicited no main effects in ROI activity. This is consistent with the comprehension results of Blanco-Elorrieta and Pylkkänen (2017), who observed that switch effects in executive control regions disappear during the comprehension of natural conversation, in contrast to single-word presentation. Our findings from minimal phrase comprehension bridge between these two findings, showing that switch effects do not emerge for short combinatory expressions either.

However, language switching did interact with orthography switching in both composition (LATL and vmPFC) and language control regions (ACC and LIFG). This was surprising in light of prior evidence that script changes incur no effects on early visual processing (Pylkkänen and Okano, 2010). Among the ROIs associated with language control, both the ACC and the LIFG elicited activity patterns, suggesting that the relationship between orthographic representation and language association is a robust variable for the bilingual brain. While switching orthographies may not affect early visual processing or lexical access, a switch in orthography seems to signal that language is also switching. This kind of signaling seems to be relevant for task completion.

From this perspective, our results align with those of other studies that suggest previously observed language-switching effects may actually be task-switching effects. The ACC and LIFG were similarly recruited for both language switching and task switching among Japanese/English bilinguals (Hosoda et al., 2012). Arabic/English bilinguals also exhibited language-switching effects in the ACC when language switches were associated with arbitrary color cues as well as defined interlocutor cues, but these effects were not observed for passive listening of naturally produced code switches (Blanco-Elorrieta and Pylkkänen, 2017). Furthermore, Mandarin/English bilinguals exhibited language-switching effects in LIFG on interlocutor cue presentation, but not when solicited for a response (Zhu et al., 2020). Abutalebi and Green (2016) argue that language-switching effects observed in the ACC and LIFG reflect mechanisms recruited for cue-based tasks such as response selection and response suppression. We add to this view by suggesting that bilinguals may use linguistic information differentially depending on the task at hand, and the ACC and LIFG play a role in managing how that linguistic information gets used.

### Conclusion

For bilinguals, language switching is natural and often the path of least resistance. Our results reveal one neural substrate that may underlie the intuitive ease of language switching: a combinatory mechanism in left anterior temporal cortex that can take mixed-language vocabulary as its input with no sensitivity to language switching. This rapid activity at 150–200 ms is followed by later prefrontal effects of language and orthography switching. Thus, it appears that the highly proficient bilingual mind represents a language switch only after a quick conceptual combination has already been performed.

## Synthesis

Reviewing Editor: Ifat Levy, Yale University School of Medicine Decisions are customarily a result of the Reviewing Editor and the peer reviewers coming together and discussing their recommendations until a consensus is reached. When revisions are invited, a fact-based synthesis statement explaining their decision and outlining what is needed to prepare a revision will be listed below. The following reviewer(s) agreed to reveal their identity: Paul Sowman.

The authors have adequately revised the paper.
